# COVID-19 and Stroke: Evaluating Correlations, Risk Factors, and Patient Outcomes

**DOI:** 10.7759/cureus.96227

**Published:** 2025-11-06

**Authors:** Hassan A Farid, Samina Alim, Fiza Shaikh, Ali R Hashim

**Affiliations:** 1 Neurology, Manchester Centre for Clinical Neurosciences, Salford, GBR; 2 Neurology, City St George's, University of London, London, GBR; 3 Neurology, University of Basrah, Basrah, IRQ

**Keywords:** cerebral venous sinus thrombosis, covid-19, cytokine storm, interleukin (il)-6, intracranial hemorrhage (ich), ischemic stroke, pandemic, stroke, stroke mortality, world pandemic

## Abstract

Coronavirus disease 2019 (COVID-19) has been associated with a range of neurological complications, including an increased risk of stroke. This study aimed to examine the relationship between COVID-19 and stroke risk in hospitalized patients. A retrospective cohort study was conducted involving 280 patients with COVID-19 and 300 non-COVID-19 controls. Sociodemographic data, medical history, COVID-19 characteristics, and inflammatory biomarkers were analyzed using chi-square tests and unpaired Student’s t-tests to assess associations with stroke occurrence. Sociodemographic factors, including age, sex, smoking status, and prevalence of chronic conditions, did not significantly differ between the two groups (p > 0.05). However, COVID-19 patients exhibited a higher prevalence of ischemic stroke (n = 17, 6.07%), hemorrhagic stroke (n = 4, 1.43%), and cerebral venous thrombosis (n = 3, 1.07%) compared to non-COVID-19 controls (p = 0.019). Severe COVID-19, prolonged illness (>2 weeks), low oxygen saturation (<70%), and cytokine storm were significantly associated with increased stroke risk (p < 0.05). Elevated interleukin-6 levels were also strongly correlated with stroke occurrence (p = 0.001). Notably, 66.7% (n = 16) of strokes occurred within the first month of infection. This study underscores the importance of considering demographic, medical, and COVID-19-related factors when assessing stroke risk in patients with COVID-19. The findings also highlight the potential role of inflammatory biomarkers, particularly interleukin-6, as indicators of stroke risk in this population.

## Introduction

Coronavirus disease 2019 (COVID-19) is a viral infectious disease caused by severe acute respiratory syndrome coronavirus 2 (SARS-CoV-2) [1]. Although mortality rates have declined since its emergence in 2019, the long-term and multisystem effects of COVID-19 continue to warrant further investigation [2,3]. Initially, COVID-19 was regarded as a disease primarily affecting the lungs [3]. In many patients, the virus caused the insidious development of pneumonia and pulmonary edema, leading to respiratory distress syndrome (RDS) [4]. However, research has since revealed its multisystem involvement, with neurological symptoms reported in up to 36% of patients [5]. Of particular interest in this study are the neurovascular manifestations, such as stroke [5].

COVID-19 has been linked to stroke development in numerous studies; however, its role as an independent risk factor remains less well understood. A case-control study reported that 46.3% of patients with COVID-19 developed acute ischemic stroke compared to 18.3% of uninfected individuals [6]. The authors concluded that COVID-19 patients should receive closer monitoring due to their increased risk [6]. Another study found that this elevated risk of ischemic stroke may persist for more than one year after infection [7]. Conversely, some studies have shown that ischemic stroke occurs primarily in COVID-19 patients with pre-existing cardiovascular risk factors [8]. Moreover, mortality risk among COVID-19 patients with stroke increases when multiple comorbidities are present [9]. A meta-analysis also identified that COVID-19 was associated with a higher risk of hemorrhagic stroke and transient ischemic attack (TIA), but only among patients with a prior history of ischemic stroke [10]. Similarly, patients with a history of hemorrhagic stroke were at greater risk of recurrence, while those with a prior TIA did not show an increased risk [10]. Rare case reports have described TIA occurring as a COVID-19 complication in patients with no prior history [11]. Takács et al. also identified a nearly 2.5-fold increase in large vessel occlusion (LVO) ischemic stroke among patients with prior COVID-19 infection compared to those without [12]. Other studies have reported associations between COVID-19 and cerebral venous sinus thrombosis (CVST), although most are limited to case reports [13,14]. A postmortem case-control study further concluded that strokes were more severe and fatal in patients with concurrent COVID-19 infection than in those without [14].

The relationship between stroke risk and the severity of COVID-19 has also been explored. Early in the pandemic, cerebrovascular events occurred in 5.7% of patients with severe COVID-19, compared to 0.8% of those with non-severe disease [5]. A meta-analysis supported this finding, identifying that severely affected patients had an approximately 3.5-fold higher risk of ischemic stroke compared to those with mild disease [15]. This may be attributed to the heightened inflammatory and coagulopathic responses observed in severe COVID-19, which are known contributors to stroke risk [5,16]. Similar mechanisms are seen in conditions such as atrial fibrillation and smoking [5]. In addition, thrombotic and hemorrhagic phenomena may coexist in COVID-19-related strokes, complicating clinical management [16]. Collectively, the evidence suggests that COVID-19 severity may serve as an independent risk factor for stroke development. Nonetheless, most existing data are derived from retrospective studies, highlighting the need for further investigation.

The null hypothesis of the present study is that hospitalized COVID-19 patients are not at increased risk of developing stroke compared to non-COVID-19 patients, and that this risk is not influenced by disease severity nor predicted by inflammatory biomarkers.

This study aimed to compare the incidence and types of stroke between COVID-19 and non-COVID-19 patients, identify demographic and clinical risk factors for stroke among COVID-19 patients, assess the association between inflammatory biomarkers and stroke occurrence, and evaluate stroke outcomes among COVID-19 patients.

## Materials and methods

This retrospective cohort study was conducted at Basrah Teaching Hospital, Basrah Governorate, Southern Iraq, during the COVID-19 pandemic.

**Figure 1:**
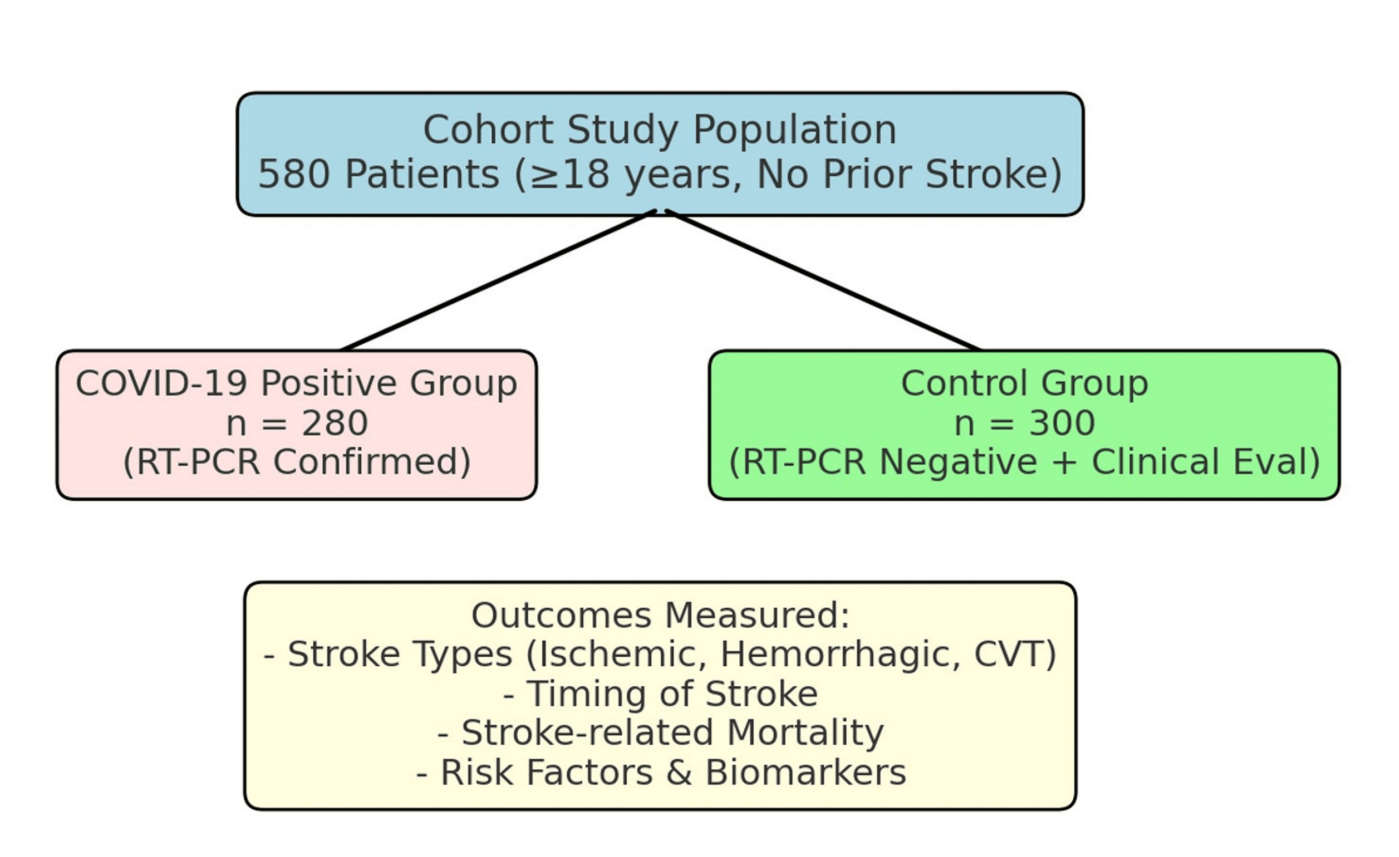
Flowchart of the study design

The study included 280 adult patients (aged ≥18 years) with confirmed severe to critical COVID-19 infection diagnosed via RT-PCR, who presented to the emergency department between January 1, 2021, and January 1, 2022. Patients with a prior history of stroke were excluded. As all eligible hospitalized COVID-19 patients within the specified period were enrolled, a formal sample size calculation was not necessary. A control group of 300 individuals with negative RT-PCR results and clinical evaluations confirming the absence of COVID-19 was also recruited (Figure 1). Controls were matched for age, sex, and vascular risk factors and were drawn from the same population cohort.

Data were collected from medical records and included the following parameters: age, sex, smoking status, and presence of chronic conditions such as hypertension, diabetes, atrial fibrillation, dyslipidemia, and ischemic heart disease. COVID-19 characteristics include disease severity, duration, lung involvement, oxygen saturation, and cytokine storm presence. The study also evaluated inflammatory biomarkers for COVID-19 cases at the time of presentation, including serum ferritin, C-reactive protein, lactate dehydrogenase, interleukin-6, and D-dimer. The types of stroke include ischemic stroke, hemorrhagic stroke, and cerebral venous thrombosis. Stroke diagnosis is established through clinical evaluation by a neurologist or stroke physician, and confirmed via imaging studies, typically a CT head or MRI, with CTV or MRV used to identify venous sinus thrombosis. The study also looked at the timing of stroke in relation to infection and the possible causes of stroke-related mortality.

Data were analyzed using descriptive and inferential statistics. Means and standard deviations were calculated for continuous variables, while categorical variables were presented as frequencies and percentages. The chi-square test was employed to assess the association between categorical variables, while the unpaired sample t-test was used to compare mean values between groups. Odds ratios were calculated to evaluate the strength of the association between COVID-19 and stroke incidence. A p < 0.05 was considered statistically significant.

## Results

Table 1 presents the sociodemographic characteristics of both COVID-19 cases and non-COVID-19 controls. Notably, the mean age and the distribution of sexes do not significantly differ between the two groups, as indicated by p-values above the significance threshold (>0.05). No substantial disparities are observed in smoking status or the prevalence of chronic conditions between the two groups. 

**Table 1:** The sociodemographic and past medical characteristics of COVID-19 cases and controls SD: standard deviation, HTN: hypertension, DM: diabetes mellitus, AF: atrial fibrillation, IHD: ischemic heart disease. ^*^Unpaired sample Student t-test. ^#^Chi-square test. Numeric data are presented as mean ± SD, and categorical data as N and %. A p < 0.05 is considered significant.

Characteristics		COVID-19 cases (n = 280)	Non-COVID-19 controls (n = 300)	p-value	Test value
Age/years	Mean ± SD	57.16 ± 12.80	56.58 ± 8.67	0.520^*^	t = 0.644
Sex	Males	161 (57.50%)	156 (52.00%)	0.183^#^	χ² = 1.77
	Females	119 (42.50%)	144 (48.00%)		
Smoking status	Smoker	58 (20.71%)	64 (21.33%)	0.854^#^	χ² = 0.03
Chronic medical illnesses	HTN	193 (68.92%)	189 (63.00%)	0.132^#^	χ² = 2.27
	DM	152 (54.29%)	155 (51.67%)	0.527^#^	χ² = 0.40
	AF	28 (10.00%)	26 (8.67%)	0.580^#^	χ² = 0.31
	Dyslipidemia	109 (38.92%)	97 (32.33%)	0.092^#^	χ² = 2.84
	IHD	46 (16.43%)	51 (17.00%)	0.853^#^	χ² = 0.04

Table 2 illustrates the association between COVID-19 and stroke types, namely, ischemic stroke, hemorrhagic stroke, and cerebral venous thrombosis. Notably, ischemic stroke demonstrates a higher prevalence in the COVID-19 group (n = 17, 6.07%) compared to the non-COVID-19 group (n = 5, 1.70%), with an odds ratio of 3.88. Similarly, hemorrhagic stroke shows an increased occurrence in the COVID-19 group (n = 4, 1.43%) compared to the non-COVID-19 group (n = 1, 0.34%), presenting an odds ratio of 4.58. Cerebral venous thrombosis exhibits a similar trend, with a higher prevalence in the COVID-19 group (n = 3, 1.07%) compared to the non-COVID-19 group (n = 1, 0.34%), yielding an odds ratio of 3.43. The obtained p-value of 0.019 from the chi-square test indicates significant differences in stroke occurrence between the COVID-19 and non-COVID-19 groups.

**Table 2:** The association between COVID-19 and the development of stroke ^#^Chi-square test. Categorical data are presented as N and %. A p < 0.05 is considered significant.

Stroke vs. COVID-19	COVID-19 cases (n = 280)	Non-COVID-19 controls (n = 300)	Odds ratio	p-value	Test value
Ischemic stroke (n = 22)	17 (6.07%)	5 (1.70%)	3.88	0.019^#^	χ² = 9.91
Hemorrhagic stroke (n = 5)	4 (1.43%)	1 (0.34%)	4.58		
Cerebral venous thrombosis (n = 4)	3 (1.07%)	1 (0.34%)	3.43		
No stroke (n = 549)	256 (91.43%)	293 (97.33%)	Reference		

Table 3 delineates the association between COVID-19 characteristics and the likelihood of experiencing a stroke. Notably, critical cases of COVID-19 are associated with an increased risk of stroke (n = 17, 12.23%). Moreover, individuals with a prolonged duration of illness, particularly beyond two weeks, exhibit a higher propensity for experiencing strokes, with a significant proportion (n = 17, 47.22%) falling into this category. Additionally, low levels of oxygen saturation, particularly below 70%, are markedly linked to an elevated risk of stroke. Furthermore, the presence of a cytokine storm, often indicative of severe inflammation, demonstrates a significant association with stroke incidence.

**Table 3:** The relationship between COVID-19 characteristics and stroke ^#^Chi-square test. Categorical data are represented as N and %. A p < 0.05 is considered significant.

COVID-19 characteristics		Stroke (n = 24)	No stroke (n = 256)	Total	p-value	Test value
Severity	Severe	7 (4.96%)	134 (95.04%)	141	0.042^#^	χ² = 6.33
	Critical	17 (12.23%)	122 (87.77%)	139		
Duration of illness	<1 week	4 (6.15%)	61 (93.85%)	65	0.001^#^	χ² = 68.7
	1-2 weeks	3 (1.68%)	176 (98.23%)	179		
	>2 weeks	17 (47.22%)	16 (52.78%)	36		
Lung involvement	<50%	1 (1.52%)	65 (98.48%)	66	0.012^#^	χ² = 6.28
	≥50%	23 (11.86%)	171 (88.14%)	194		
O₂ saturation	70-93%	5 (3.16%)	153 (96.84%)	158	0.001 ^#^	χ² = 11.2
	<70%	18 (14.75%)	104 (85.25%)	122		
Cytokine storm	Present	23 (13.53%)	147 (86.47%)	170	0.001 ^#^	χ² = 14.3
	Absent	1 (0.91%)	109 (99.09%)	110		

Table 4 illustrates the association between inflammatory biomarkers and the incidence of stroke. Despite variations in mean levels between individuals with stroke and those without, none of the biomarkers, serum ferritin, C-reactive protein, lactate dehydrogenase, or D-dimer, show a statistically significant difference in their levels. However, it is noteworthy that interleukin-6 levels display a notable contrast between stroke and non-stroke groups, which is statistically significant (p = 0.001).

**Table 4:** The relationship between the inflammatory biomarker levels and stroke ^*^Unpaired sample Student t-test. Numeric data are presented as mean ± SD. A p < 0.05 is considered significant.

Biomarker level (mean ± SD)	Stroke (n = 24)	No stroke (n = 256)	p-value	Test value
Serum ferritin (mcg/L)	1477.70 ± 619.06	1393.20 ± 499.56	0.517^*^	t = 0.65
C-reactive protein (mg/L)	70.27 ± 32.50	63.70 ± 18.95	0.331^*^	t = 0.97
Lactate dehydrogenase (U/L)	672.25 ± 303.78	647.73 ± 314.03	0.714^*^	t = 0.37
Interleukin-6 level (pg/mL)	401.21 ± 113.82	249.10 ± 170.05	0.001^*^	t = 4.11
D-dimer (ng/mL)	2862.30 ± 536.30	2577.40 ± 480.00	0.480^*^	t = 0.71

**Figure 2:**
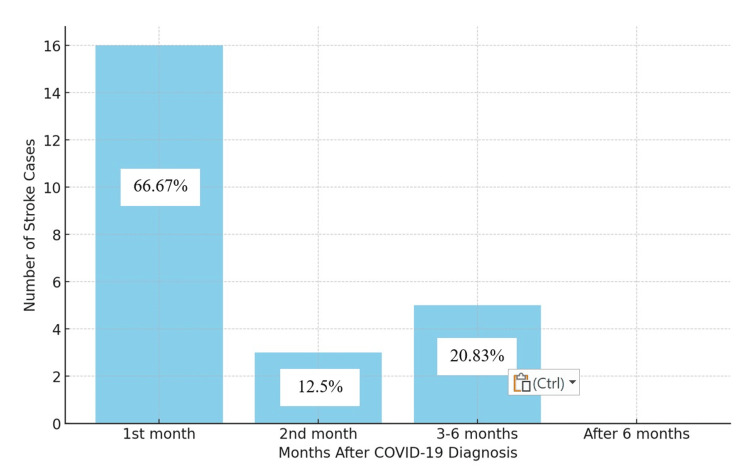
Timing of stroke occurrence

Figure 2 provides a clear representation of the temporal distribution of stroke cases following COVID-19, illustrating the stroke occurrences among 24 patients post-COVID-19, with the majority (n = 16, 66.67%) experiencing a stroke within the first month, a few (n = 3, 12.5%) in the second month, and others (n = 5, 20.83%) between three and six months after their COVID-19 diagnosis. No strokes were reported after six months.

**Figure 3:**
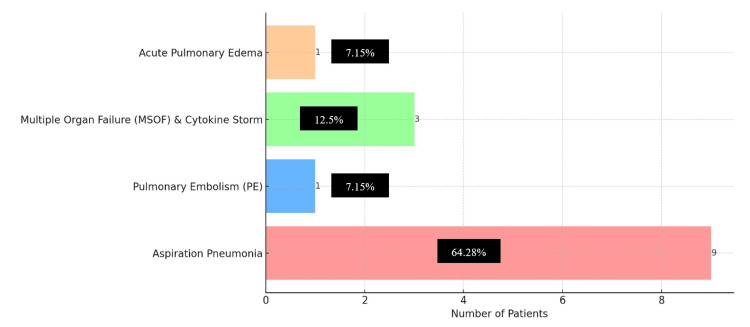
Causes of death among stroke patients

Figure 3 shows the causes of death among stroke patients post-COVID-19. Most deaths (9/14) (64.28%) were due to aspiration pneumonia, followed by multiple organ failure and cytokine storm (3/15) (12.5%), pulmonary embolism (1/14) (7.15%), and acute pulmonary edema (1/14) (7.14%).

## Discussion

Numerous studies have demonstrated an association between stroke and COVID-19. A systematic review and meta-analysis from 2022 reported a potential stroke risk of 2% among COVID-19 patients of all ages [17]. Cryptogenic stroke was the most common subtype, with a pooled prevalence of 35% [17]. Another study showed a threefold higher incidence of stroke in patients with severe COVID-19 compared to those with mild or moderate disease, although the pooled risk among hospitalized COVID-19 patients was lower at 0.9% [18]. Similar findings have been observed across other studies. Although the exact mechanism remains unclear, proposed explanations include amplified inflammatory responses, prothrombotic states, endothelial injury, and plaque instability. Some studies suggest that coronavirus binds to angiotensin-converting enzyme-2 (ACE-2) receptors in central nervous system (CNS) cells, initiating a cascade that promotes hypertension, vasoconstriction, and thrombosis [19-21]. Others emphasize cytokine-driven endothelial damage and hypercoagulability [18]. Elevated D-dimer and platelet levels, frequently seen in critical COVID-19 cases, may further contribute to acute cerebrovascular events [19]. The results of our study align with existing literature [18-21], reinforcing the association between COVID-19 and an increased risk of stroke, particularly ischemic stroke. As shown in Table 2, there was a statistically significant difference in stroke prevalence between the COVID-19 and non-COVID-19 groups, with odds ratios indicating a higher risk in the COVID-19 cohort. Specifically, ischemic stroke occurred more frequently in COVID-19 patients, consistent with previous findings [18], suggesting that severe infection elevates stroke risk through inflammation, endothelial dysfunction, and hypercoagulability [19-21]. These mechanisms are supported by our results in Table 3, where severe COVID-19, prolonged illness duration, and reduced oxygen saturation were significantly associated with stroke incidence.

Inflammation plays a central role in the pathogenesis of stroke. Experimental data show that inflammation is involved in every stage of atherosclerosis, from plaque formation to rupture, leading to neurovascular events [22]. A 2023 study found elevated levels of serum ferritin, CRP, D-dimer, and IL-6 in post-stroke patients: CRP was elevated in 100%, D-dimer in 87%, and ferritin and IL-6 in 60% [23]. IL-6, one of the most widely studied inflammatory markers, has been identified as a strong predictor of stroke risk; baseline IL-6 levels above 5 pg/mL increase the risk of vascular events 21-fold in patients with prior stroke who are not anticoagulated [24]. CRP, an acute-phase reactant produced under IL-6 stimulation, is another well-established biomarker associated with stroke [25,26], with levels above 3.0 mg/L tripling stroke risk [27]. Similar associations have been observed with high-sensitivity CRP (hs-CRP) [28]. Conversely, research on LDH as a stroke biomarker has produced inconsistent results. A 2024 Mendelian randomization study suggested an inverse causal relationship, whereas earlier observational studies reported a positive association [29,30]. In our study, only interleukin-6 (IL-6) showed a significant difference between stroke and non-stroke groups. Elevated IL-6 has been consistently linked to stroke risk [24], and its association in our findings highlights the potential utility of inflammatory markers in predicting cerebrovascular complications among COVID-19 patients [23-28]. Although other markers such as CRP and D-dimer did not reach statistical significance, their elevated patterns suggest that inflammation-driven pathways may underlie the increased stroke risk in COVID-19, consistent with prior research.

Overall, our study supports existing evidence linking COVID-19 with an increased risk of stroke and highlights the clinical and inflammatory factors that may influence this risk. The trends observed in biomarker profiles and clinical characteristics of COVID-19 patients who developed stroke offer valuable insights into possible predictive mechanisms. Further research is warranted to clarify these associations and to develop targeted preventive strategies for at-risk populations.

This study has several limitations. As a single-center study conducted in Basrah, Iraq, the findings may not be generalizable to other settings with differing demographics or COVID-19 severities. The retrospective design relies on medical records, which may introduce biases such as incomplete or inaccurate documentation and limit control over confounding variables. Although known risk factors were accounted for, unmeasured confounders, such as genetic predisposition, vaccination status, or socioeconomic factors, may have influenced the observed associations. Furthermore, the 12-month study period captures short-term stroke risk but does not address potential long-term neurological consequences following COVID-19 infection.

## Conclusions

This study demonstrates a significantly higher risk of stroke, particularly ischemic and hemorrhagic, in patients with COVID-19 compared to non-COVID-19 controls. Critical illness, prolonged disease duration, low oxygen saturation, and cytokine storm were identified as key contributing factors. Among inflammatory markers, only IL-6 showed a significant association with stroke occurrence. No significant differences were observed in sociodemographic characteristics between the two groups. Clinicians should remain vigilant in monitoring these high-risk features among COVID-19 patients to facilitate early detection and reduce stroke incidence.

## References

[1] Shi Y, Wang G, Cai XP (2020). An overview of COVID-19. J Zhejiang Univ Sci B.

[2] Di Toro A, Bozzani A, Tavazzi G (2021). Long COVID: long-term effects?. Eur Heart J Suppl.

[3] Vosko I, Zirlik A, Bugger H (2023). Impact of COVID-19 on cardiovascular disease. Viruses.

[4] Xu Z, Shi L, Wang Y (2020). Pathological findings of COVID-19 associated with acute respiratory distress syndrome. Lancet Respir Med.

[5] Zakeri A, Jadhav AP, Sullenger BA, Nimjee SM (2021). Ischemic stroke in COVID-19-positive patients: an overview of SARS-CoV-2 and thrombotic mechanisms for the neurointerventionalist. J Neurointerv Surg.

[6] Belani P, Schefflein J, Kihira S (2020). COVID-19 is an independent risk factor for acute ischemic stroke. AJNR Am J Neuroradiol.

[7] Eberhardt N, Noval MG, Kaur R (2023). SARS-CoV-2 infection triggers pro-atherogenic inflammatory responses in human coronary vessels. Nat Cardiovasc Res.

[8] Qureshi AI, Baskett WI, Huang W (2021). Acute ischemic stroke and COVID-19: an analysis of 27 676 patients. Stroke.

[9] Gofir A, Satriotomo I, Syamsah YC (2024). Degree of COVID-19 severity and mortality in stroke: correlation of clinical and laboratory parameters. BMC Neurosci.

[10] Nia AM, Srinivasan VM, Lall RR, Kan P (2022). COVID-19 and stroke recurrence by subtypes: a propensity-score matched analyses of stroke subtypes in 44,994 patients. J Stroke Cerebrovasc Dis.

[11] Singh J, Robinson S (2022). An interesting case of COVID-19 with transient ischemic attack as a delayed neurological complication. J Family Med Prim Care.

[12] Algarni SA, ALhasab NS, Alharbi MS, Albarrak A, Alanezi AA, Al Shehri HM (2024). Sex differences and clinical outcomes of patients with coronavirus disease 2019 infection and cerebral venous sinus thrombosis: a systematic review. Clin Appl Thromb Hemost.

[13] Takács TT, Berki ÁJ, Böjti PP (2023). The impact of SARS-CoV-2 infection on the outcome of acute ischemic stroke-a retrospective cohort study. PLoS One.

[14] Immovilli P, Marchesi E, Terracciano C (2022). A "post-mortem" of COVID-19-associated stroke: a case-control study. J Stroke Cerebrovasc Dis.

[15] Lu Y, Zhao JJ, Ye MF, Li HM, Yao FR, Kong Y, Xu Z (2021). The relationship between COVID-19's severity and ischemic stroke: a systematic review and meta-analysis. Neurol Sci.

[16] Katz JM, Libman RB, Wang JJ (2021). COVID-19 severity and stroke: correlation of imaging and laboratory markers. AJNR Am J Neuroradiol.

[17] de Souza AM, de Araújo EF, Junior NC, Raimundo AC, Pereira AC, de Castro Meneghim M (2025). Association between SARS-CoV-2 and stroke: perspectives from a metaumbrella-review. BMC Neurol.

[18] Shahjouei S, Naderi S, Li J (2020). Risk of stroke in hospitalized SARS-CoV-2 infected patients: A multinational study. EBioMedicine.

[19] Qin C, Zhou L, Hu Z (2020). Dysregulation of immune response in patients with coronavirus 2019 (COVID-19) in Wuhan, China. Clin Infect Dis.

[20] Wu Y, Xu X, Chen Z (2020). Nervous system involvement after infection with COVID-19 and other coronaviruses. Brain Behav Immun.

[21] Xia H, Sriramula S, Chhabra KH, Lazartigues E (2013). Brain angiotensin-converting enzyme type 2 shedding contributes to the development of neurogenic hypertension. Circ Res.

[22] Kelly PJ, Lemmens R, Tsivgoulis G (2021). Inflammation and stroke risk: a new target for prevention. Stroke.

[23] Jumagaliyeva MB, Ayaganov DN, Abdelazim IA, Saparbayev SS, Tuychibaeva NM, Kurmambayev YJ (2023). Acute cerebrovascular events and inflammatory markers associated with COVID-19: An observational study. J Med Life.

[24] Cao Y, Yue X, Jia M, Wang J (2023). Neuroinflammation and anti-inflammatory therapy for ischemic stroke. Heliyon.

[25] Bustamante A, Simats A, Vilar-Bergua A, García-Berrocoso T, Montaner J (2016). Blood/brain biomarkers of inflammation after stroke and their association with outcome: from C-reactive protein to damage-associated molecular patterns. Neurotherapeutics.

[26] Kaptoge S, Di Angelantonio E, Lowe G (2010). C-reactive protein concentration and risk of coronary heart disease, stroke, and mortality: an individual participant meta-analysis. Lancet.

[27] Ballantyne CM, Hoogeveen RC, Bang H (2005). Lipoprotein-associated phospholipase A2, high-sensitivity C-reactive protein, and risk for incident ischemic stroke in middle-aged men and women in the Atherosclerosis Risk in Communities (ARIC) study. Arch Intern Med.

[28] Dawood FZ, Judd S, Howard VJ (2016). High-sensitivity C-reactive protein and risk of stroke in atrial fibrillation (from the reasons for geographic and racial differences in stroke study). Am J Cardiol.

[29] Dong F, Wang X, Li J, Zhao D, Li J (2024). Causal relationship between lactate dehydrogenase and risk of developing ischemic stroke: a Mendelian randomized study. Brain Behav.

[30] Wang A, Tian X, Zuo Y (2021). High lactate dehydrogenase was associated with adverse outcomes in patients with acute ischemic stroke or transient ischemic attack. Ann Palliat Med.

